# Expression of survivin and its splice variants survivin-2B and survivin-ΔEx3 in breast cancer

**DOI:** 10.1038/sj.bjc.6602314

**Published:** 2004-12-21

**Authors:** B Ryan, N O'Donovan, B Browne, C O'Shea, J Crown, A D K Hill, E McDermott, N O'Higgins, M J Duffy

**Affiliations:** 1 Department of Surgery, Conway Institute of Biomolecular and Biomedical Research, University College Dublin, Dublin 4, Ireland; 2 Department of Medical Oncology, St Vincent's University Hospital, Dublin 4, Ireland; 3 Department of Nuclear Medicine, St Vincent's University Hospital, Dublin 4, Ireland

**Keywords:** inhibitor of apoptosis, apoptosis, programmed cell death, breast carcinoma

## Abstract

Alternative splicing of survivin mRNA gives rise to multiple isoforms, that is, survivin and 3 splice variants, survivin-2B, survivin-3B and survivin-ΔEx3. The aim of this study was to compare the expression of survivin, survivin-2B and survivin-ΔEx3 in normal breast tissue, fibroadenomas, primary breast cancer and axillary nodal metastases. Survivin, survivin-2B and survivin-ΔEx3 mRNA were measured using semiquantitative RT–PCR. In the primary carcinomas, we related mRNA for each form of survivin to both survivin protein and apoptosis. For each type of breast tissue, survivin was the predominant form detected, being present in 146 out of 156 (93.6%) primary breast carcinomas, 11 out of 11 (100%) axillary nodal metastases, 21 out of 31 (67.7%) fibroadenomas and five out of 22 (22.7%) specimens of normal breast tissue. Levels of the three forms of survivin were significantly higher in the carcinomas compared to normal breast tissue (*P*<0.0001). Levels of both survivin-2B and survivin-ΔEx3 but not survivin were significantly higher in nodal metastases than primary carcinomas. Survivin mRNA levels correlated significantly with survivin protein. Finally, both survivin and survivin-ΔEx3 but not survivin-2B correlated positively with apoptosis. Although survivin, survivin-2B and survivin-ΔEx3 were all detected in both malignant and nonmalignant breast tissue, the predominant form was survivin. Our results suggest that the different forms of survivin may have different roles in apoptosis in breast cancer.

Survivin is a member of the inhibitor of apoptosis (IAP) family of molecules and is involved in both inhibition of apoptosis and regulation of cell division. Although rarely expressed in terminally differentiated normal adult tissues, survivin is upregulated in most malignancies (Reed, 2001; Altieri, 2003). For example, SAGE analysis of the expression profile of 3.5 million transcripts in diverse cancer types identified survivin as one of the top four transcripts upregulated in malignancy (Velculescu *et al*, 1999). These original findings using SAGE analysis have now been confirmed in multiple cancer types using standard techniques such as immunohistochemistry and RT–PCR (Altieri, 2003). This upregulation combined with its dual role in apoptosis and cell division suggests that survivin plays a critical role in cancer.

In addition to the original survivin transcript described by Ambrosini *et al* (1997), three survivin splice variants have been identified (Mahotka *et al*, 1999; Badran *et al*, 2004). Survivin-2B has an additional exon of 69 base pairs, while survivin-ΔEx3 lacks 118 base pairs of exon 3 (Mahotka *et al*, 1999). Survivin-3B, which was only recently identified, contains five exons including exon 3B derived from a 165-base pair long portion of intron 3 (Badran *et al*, 2004). In transfection experiments, survivin-ΔEx3 demonstrated similar antiapoptotic potential to survivin but survivin-2B exhibited reduced antiapoptotic potential (Mahotka *et al*, 1999). A possible antiapoptotic role for survivin 3B remains to be determined. In contrast to full-length survivin, relatively little work has been performed on expression of the survivin splice variants in tumour tissues.

The aim of this study was therefore to examine expression of survivin, survivin-2B and survivin-ΔEx3 mRNA in a large panel of primary breast carcinomas, axillary nodal metastases from primary breast cancers, fibroadenomas and normal breast tissues. In the primary carcinomas, we related mRNA for the three forms of survivin to tumour histopathological characteristics, hormone receptors, survivin protein and levels of apoptosis.

## MATERIALS AND METHODS

### Sample processing

Breast carcinomas, breast fibroadenomas, normal breast tissue samples and nodal metastases from breast cancer patients were obtained at the time of surgery, snap frozen in liquid nitrogen and stored at −80°C. Normal breast tissues were obtained from reduction mammoplasty tissues (*n*=6), normal tissue adjacent to fibroadenomas (*n*=2) and normal tissue adjacent to carcinomas (*n*=14). Although these tissues are referred to as ‘normal’ breast tissues, it is important to point out that they cannot be regarded as ‘healthy normal’ specimens. A detailed description of the primary breast carcinomas used is shown in Table 1. Tissue samples were homogenised using a Mikro-Dismembrator U (Braun Biotech Intl., Melsungen, Germany) to yield a fine powder. An aliquot of the powder was extracted with 50 mM Tris buffer (pH 7.4) containing 1 mM monothioglycerol and assayed for oestrogen receptor (ER) and progesterone receptor (PR) by ELISA (Abbott Diagnostics, N Chicago, IL, USA). The cutoff points for ER and PR were 200 fmol g^−1^ wet weight tissue and 1000 fmol g^−1^ wet weight tissue, respectively.

### RNA extraction

RNA extractions were performed using an RNace Total Pure kit (Bioline, Randolph, MA, USA). RNA integrity was assessed by gel electrophoresis and concentrations were measured spectrophotometrically.

### RT–PCR analysis

cDNA was synthesized from 1 *μ*g of total RNA, using 50 *μ*M oligo (dT)_12–18_ primers (Promega, Madison, WI, USA), 0.4 mM dNTPs (Promega), 1 × MMLV buffer and 100 U of MMLV reverse transcriptase (Invitrogen Life Technologies, Carlsbad, CA, USA). PCR was performed using primers which amplify the various survivin splice variants (forward: 5′-GCA TGG GTG CCC CGA CGT TG-3′, reverse: 5′-GCT CCG GCC AGA GGC CTC AA-3′). GAPDH was used as an internal control (forward: 5′-GCC TCA AGA TCA TCA GCA A-3′, reverse: 5′- CCA GCG TCA AAG GTG GAG-3′). Briefly, the 25 *μ*l reaction mix contained 1 *μ*l cDNA, 250 *μ*M dNTPs, 50 ng of each primer, *Taq* polymerase buffer (10 mM Tris-HCl (pH 9.0), 50 mM KCl, 0.1% Triton®X-100 (TBS-T)) and 1.25 U *Taq* polymerase (Promega). Amplification conditions were as follows: 2 min at 94°C, followed by 30 cycles of 30 s at 94^o^C, 1 min at 62°C and 1 min at 72°C with a final extension of 5 min at 72°C. The PCR products were visualised on a 2% agarose gel. Band intensities for all visible bands were measured by densitometry using the Eagle Eye gel documentation system (Stratagene, La Jolla, CA, USA) and expressed as arbitrary units, relative to GAPDH. The three survivin PCR products were gel purified using a DNA extraction kit (Qiagen, Valencia, CA, USA) and sequenced on an ABI Prism 310 instrument to verify the identity of the bands.

### Detection of survivin by Western blotting

Tissue cytosols were diluted in sodium dodecyl sulphate (SDS) buffer containing 50 mM Tris-HCL (pH 6.8), 5% beta mercaptoethanol, 2% SDS, 8% glycerol and 0.01% bromophenol blue and heated to 95°C for 5 min. In all, 200 *μ*g of cytosolic protein extract from each sample was separated on a 15% SDS–polyacrylamide gel. Recombinant survivin, generously donated by Dr K Shiraki, Mie University School of Medicine, Mie, Japan, was included in each experiment as a positive control. Following electrophoresis, the separated proteins were transferred to a nitrocellulose membrane (Sigma, St Louis, MO, USA) using a semi-dry blotting apparatus for 45 min. The membranes were then treated for 1 h at room temperature with a blocking solution containing 50 mM Tris-buffered saline (TBS), 5% skimmed milk powder and 0.05% Triton-X-100 (TBS-T) (Sigma, St Louis, MO, USA). Following this, the membranes were incubated overnight at 4°C with mouse monoclonal anti-survivin (1 : 1000) (Novus Biologicals, Littleton, CO, USA), in a blocking solution containing 50 mM TBS-T and 1% skimmed milk powder. Subsequently, membranes were washed in 50 mM TBS containing 0.05% Triton and then probed with horseradish peroxidase (HRP)-conjugated goat anti-mouse secondary antibody (Sigma). The blots were washed three times for 10 min each in 50 mM TBS-T followed by a 10 min wash in 50 mM TBS. Immunoreactivity was detected using enhanced chemiluminescence (ECL) reagent (Luminol, Santa Cruz, CA, USA). As a control for equal loading, membranes were reprobed with mouse anti-*β*-actin antibody (Sigma, St Louis, MO, USA). Protein levels were quantified using densitometry on an Eagle Eye gel documentation system (Stratagene, La Jolla, CA, USA).

### Measurement of apoptosis

Apoptosis was measured by the Cell Death Detection ELISA (Roche, Mannheim, Germany) as previously described (O'Donovan *et al*, 2003). Briefly, 30 *μ*g of cytosolic extract from each sample was incubated in 96-well microtitre plates coated with biotin-conjugated anti-histone antibody for 1 h. After four washes, samples were incubated with biotin-conjugated anti-DNA antibody for 1 h at room temperature. ABTS (2,2′-azino-bis-(3-benzthiazoline-6-sulfonic acid)) solution was used for colorimetric detection. Absorbances were measured at 405 nm and at a reference wavelength of 490 nm. A nucleosome-positive control included in the kit was used in each assay.

### Statistical analysis

Nonparametric Spearman Rank correlations, Mann–Whitney *U*-tests and *χ*^2^ tests were performed using StatView 5.0.1 (SAS Institute Inc., Cary, NC, USA). A *P*-value of <0.05 was regarded as statistically significant.

## RESULTS

### Expression of survivin variants in nonmalignant and malignant breast tissue

**Figure 1:**
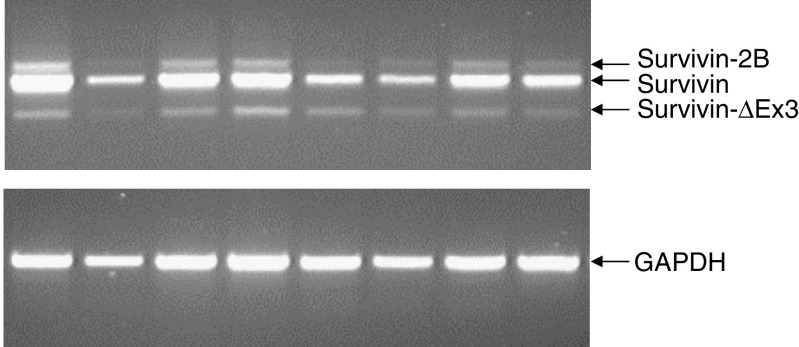
RT–PCR products of survivin, survivin-2B and survivin-ΔEx3 in eight representative primary breast carcinoma samples. The identity of each band was confirmed by sequencing.

Following RT–PCR, three different bands were seen (Figure 1). Based on sequence analysis, the three bands corresponded to survivin, survivin-2B and survivin-ΔEx3. Table 2 summarises the distribution of the three forms of survivin in normal breast tissue, fibroadenomas, primary breast carcinomas and axillary nodal metastases from primary breast cancers. For each type of breast tissue investigated, survivin was the predominant form detected. Survivin was detected in 93.6% (146 out of 156) of primary breast carcinomas and in all of the 11 axillary nodal metastases investigated compared to 22.7% (5 out of 22) of normal breast tissues and 67.7% (21 out of 31) of fibroadenomas. Of the five normal breast tissues, positive for survivin, three were from tissue remote from a carcinoma while two were from reduction mammoplasty specimens. The two splice variants, survivin-2B and survivin-ΔEx3, were only detected in one out of 22 (4.5%) normal breast tissue sample. Levels of the three forms of survivin were significantly higher in the primary carcinomas than in the normal breast specimens (*P*<0.0001 for each form). Levels of survivin-2B and survivin-ΔEx3 were significantly higher in the nodal metastases than in the primary cancers (for survivin-2B, *P*=0.0007; for survivin-ΔEx3, *P*=0.02) (Table 2).

In the primary carcinomas expression of survivin correlated strongly with survivin-2B and survivin-ΔEx3 (for survivin-2B, *P*<0.0001, *r*=0.454; for survivin-ΔEx3, *P*<0.0001, *r*=0.617). In addition, expression of the 2B variant correlated with expression of the ΔEx3 variant (*P*<0.0001, *r*=0.728). The survivin-2B and survivin-ΔEx3 splice variants were not detected in any sample lacking survivin.

### Relationship between survivin and characteristics of the primary cancers

Table 3 summarises the relationship between survivin, survivin-2B and survivin-ΔEx3 and characteristics of the primary cancers. No significant correlation was found between any of the survivin forms investigated and tumour grade, ER or PR status. Levels of survivin-ΔEx3 showed a weak inverse relationship with tumour size (*r*=−0.16, *P*=0.017, *n*=148) and numbers of nodal metastases (*r*=−0.10, *P*=0.042, *n*=148). Levels of survivin-ΔEx3 were also significantly higher in ductal compared to lobular cancers (*P*=0.0388, *n*=140) (Table 3).

### Relationship between survivin splice variants and survivin protein

Survivin protein was measured by Western blotting in 73 primary carcinomas. The survivin antibody detected a single band of 16.5 kDa. Survivin protein was detected in 86.6% (63 out of 73) of the primary breast carcinomas. Levels of full-length survivin mRNA correlated with survivin protein (*r*=0.48, *P*=0.041, *n*=73). Of the 73 cancers, both survivin mRNA and protein were detected in 64 (87.7%) and was negative for both in two (2.7%) of the samples. Seven (9.6%) were positive at the mRNA level but negative at the protein level. Survivin protein was not found in any sample lacking detectable survivin mRNA. Survivin protein did not correlate with the levels of either survivin-2B or survivin-ΔEx3 (for survivin-2B, *r*=0.21, *P*=0.324; for survivin-ΔEx3, *r*=0.19, *P*=0.259).

### Relationship between survivin variants and apoptosis in primary carcinomas

**Figure 2:**
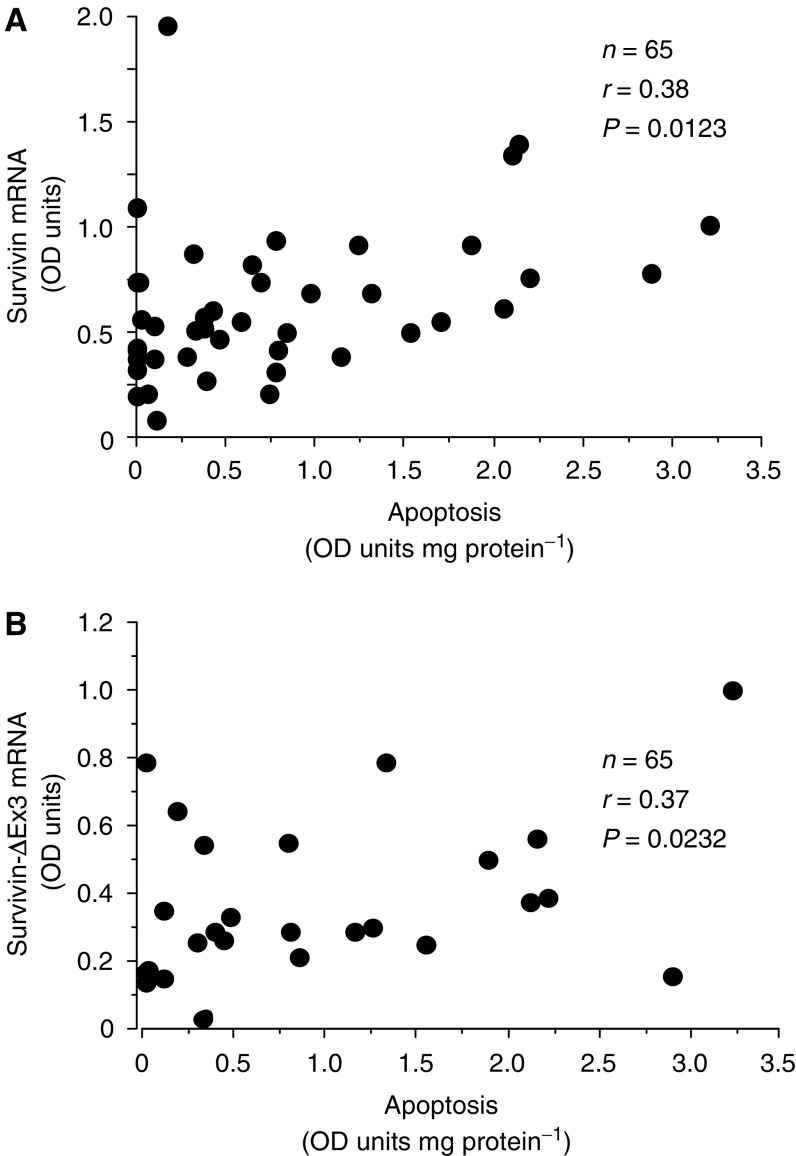
Relationship between both survivin mRNA (**A**) and survivin-ΔEx3 mRNA (**B**) and apoptosis levels in primary breast cancer. OD units are optical density units.

Survivin has been shown to inhibit apoptosis but the relationship between the survivin splice variants and apoptosis has not previously been investigated in breast cancer. Levels of apoptosis were measured in 65 of the primary carcinoma samples using the Cell Death Detection ELISA, which measures nucleosomes released during DNA fragmentation. As shown in Figures 2A and B, levels of both survivin and survivin-ΔEx3 mRNA showed a moderate positive correlation with apoptosis (for survivin, *r*=0.38, *P*=0.012; for survivin-ΔEx3, *r*=0.37, *P*=0.023). In contrast, no significant correlation was found between survivin-2B and apoptosis (*n*=65, *r*=0.28, *P*=0.121).

## DISCUSSION

Although expression of survivin has been widely studied in cancer (Altieri, 2003; Li, 2003), there are relatively few reports on its splice variants. Indeed to our knowledge, only one previous study has investigated survivin splice variants in breast cancer. In 2003, O'Driscoll *et al* reported that survivin, survivin-ΔEx3 and survivin-2B were present in 68, 55 and 9.4% of breast cancers, respectively. Compared to O'Driscoll *et al* (2003), we found that all of the three forms were detected in a higher proportion of carcinomas. This difference was particularly marked for survivin-2B and may relate to the fact that different PCR procedures were used in the two studies. Although the same survivin primers were used in both studies, there were differences in the protocol. In particular, the use of an abundant transcript, *β*-actin, as an endogenous PCR control may explain differences in the sensitivity of detection of less-abundant transcripts such as the survivin splice variants.

Similar to previous studies in both gastric (Krieg *et al*, 2002) and renal carcinomas (Mahotka *et al*, 2002a) as well as breast cancer (O'Driscoll *et al*, 2003), we show here that full-length survivin is also the main form of survivin expressed in normal breast tissue, fibroadenomas and breast carcinomas.

Survivin, survivin-2B and survivin-ΔEx3 mRNAs were detected at significantly lower frequencies and lower levels in the normal breast tissue compared to the primary breast carcinomas. Levels of the two splice variants but not full-length survivin were significantly higher in nodal metastases compared to the primary breast cancers.

Although early studies showed little or no expression of survivin in normal differentiated tissues (Adida *et al*, 1998; Altieri 2003; Li 2003), we found that survivin was detected in five out of 22 samples of normal breast tissue and 21 out of 31 fibroadenomas. The splice variants however, were detected in only one of the 22 normal breast tissues tested, that is, in a sample remote from a carcinoma. Consistent with our data, Hirohashi *et al* (2002) failed to detect the splice variants in a number of normal adult tissues. These findings suggest that survivin-2B and survivin-ΔEx3 may be more specific markers for malignancy than survivin itself. Although survivin mRNA levels were significantly higher in primary breast cancer compared to fibroadenomas, survivin was detected in greater than half of the fibroadenomas investigated, which suggests that survivin may play a role in the development of these benign tumours.

In agreement with previous studies (Tanaka *et al*, 2000; Nasu *et al*, 2002; Kennedy *et al*, 2003), we found no significant correlation between survivin and tumour size, tumour grade, nodal status, histology type or hormone receptor status. However, a weak but significant inverse relation was found between survivin-ΔEx3 and both tumour size and number of nodal metastases. This form of survivin was also detected more frequently in ductal compared to lobular primary carcinomas. Previously, we reported that caspase 3 levels were also significantly higher in ductal than lobular breast cancers (O'Donovan *et al*, 2003). These findings suggest that the regulation of apoptosis is different in ductal and lobular breast cancers.

Although the number of nodal metastases investigated in this study was relatively small, that is, 11, all samples were positive for survivin. If this preliminary result can be confirmed with larger numbers of samples, survivin could be a sensitive marker for detecting micrometastases in lymph nodes from breast cancer patients. In this context, it should be pointed out that in melanoma, patients with survivin-positive sentinel lymph nodes had a significantly worse outcome than those with survivin-negative nodes (Gradilone *et al*, 2002).

Survivin protein correlated significantly with survivin mRNA in the 73 primary carcinomas tested. The antibody used for Western blotting was a monoclonal antibody raised against full-length recombinant survivin protein and can be blocked using a survivin C-terminus peptide (amino acids 129–142). Omission of exon 3 in survivin-ΔEx3 causes a frame-shift in exon 4, which results in an altered C-terminus in the predicted survivin-ΔEx3 protein. Therefore, the antibody used would not be expected to detect survivin-ΔEx3 protein. Survivin-2B includes amino acids 129–142 of survivin and the predicted molecular weight of the survivin-2B protein is 18.6 kDa. However, we did not observe any bands of 18.6 kDa. A single band of 16.5 kDa, which is the expected size for survivin protein, was observed in all samples positive for survivin protein.

In this study, we showed that levels of both survivin and survivin-ΔEx3 but not survivin-2B correlated significantly with apoptosis. As mentioned in the introduction, Mahotka *et al* (1999) reported that survivin and survivin-ΔEx3 were antiapoptotic, whereas survivin-2B exhibited a reduced antiapoptotic ability. Indeed, it has been suggested that that the 2B variant might be a naturally occurring antagonist of survivin and survivin-ΔEx3, possibly by competitive binding to a common interaction partner (Mahotka *et al*, 1999, 2002b). Why survivin and survivin-ΔEx3 correlate positively with apoptosis is unclear. A possible explanation, however, is that upregulation of survivin expression occurs in response to the increased caspase levels and/or rates of apoptosis, which is found in breast cancer (Krajewski *et al*, 1999; Mommers *et al*, 1999; Zhao *et al*, 2002; O'Donovan *et al*, 2003). It should be pointed out that Sarela *et al* (2002), in a study on pancreatic cancer, also found a significant and positive correlation between survivin mRNA and apoptosis. In other reports, however, either an inverse relationship (Kawasaki *et al*, 2001; Satoh *et al*, 2001) or no significant relationship has been found between survivin and rates of apoptosis in tumours (Beardsmore *et al*, 2003).

While this work was in progress, a new variant of survivin, that is, survivin-3B was described (Badran *et al*, 2004). Survivin-3B comprises five exons, including a novel exon 3B of 165 base pairs derived from intron 3. As survivin-3B contains a BIR domain, it is likely to participate in apoptosis. The survivin primers used in this study should detect survivin-3B as a PCR product 165 base pairs larger than survivin. A band of that size was however, not observed using conventional gel electrophoresis. This may mean that it is not expressed in breast tissue or that our assay may not be sensitive enough to detect it. The presence or absence of this splice variant in breast tissue could be confirmed by PCR with specific primers for the 3B variant as described by Badran *et al* (2004).

Clearly, further work is necessary to establish the biological role for the different forms of survivin. In order to address a potential clinical value for the three forms of survivin investigated in this study, we will, in the future, relate each survivin form to both patient outcome and response to therapy.

## Figures and Tables

**Table 1:** Characteristics of primary carcinomas used

**Characteristic**	***n***	**%**
*Size (cm)*		
⩽2	34	21.8
>2	114	73.1
Unknown	8	5.1
*Grade*		
1+2	57	36.5
3	77	49.4
Unknown	22	14.1
*Nodal status*		
Positive	85	54.5
Negative	63	40.4
Unknown	8	5.1
*ER status*		
Positive	102	65.4
Negative	41	26.3
Unknown	13	8.3
*PR status*		
Positive	56	35.9
Negative	90	57.7
Unknown	10	6.4
*Histology*		
Ductal (D)	115	73.7
Lobular (L)	25	16.0
Mixed D+L	11	7.1
Unknown	5	3.2

**Table 2:** Frequency and median levels of survivin, survivin-2B and survivin-ΔEx3 mRNA in normal breast tissue, fibroadenomas, primary breast cancer and axillary nodal metastases from breast cancer

	**Survivin**		**Survivin-2B**		**Survivin-ΔEx3**	
**Tissue**	**No. positive (%)**	**Median level**	**No. positive (%)**	**Median level**	**No. positive (%)**	**Median level**
Normal breast (*n*=*22*)	5 (22.7)	0	1 (4.5)	0	1 (4.5)	0
Fibroadenoma (*n*=*31*)	21 (67.7)	0.118	15 (48.4)	0	16 (51.6)	0
Primary breast carcinoma (*n*=*156*)	146 (93.6)	0.467	96 (61.5)	0.041	86 (55.1)	0.025
Nodal metastases (*n*=*11*)	11 (100)	0.509	9 (81.8)	0.255	5 (45.5)	0.197

**Table 3:** Relationship between survivin, survivin-2B and survivin-ΔEx3 and established prognostic factors in breast cancer

		**Median levels (OD units/relative to GAPDH)**		
**Characteristic**	***n***	**Survivin**	**Survivin-2B**	**Survivin-ΔEx3**
*Size (cm)*				
⩽2	34	0.488	0.096	0.134
>2	114	0.523	0.110	0.142
				
*Nodal status*				
Positive	85	0.490	0.113	0.118
Negative	63	0.534	0.103	0.157
				
*Histology*				
Ductal(D)	115	0.549	0.112	0.146*
Lobular (L)	25	0.386	0.075	0.084
Mixed D+L	11	0.405	0.114	0.159
				
*Grade*				
1+2	57	0.495	0.104	0.133
3	77	0.568	0.124	0.154
				
*ER status*				
Positive	102	0.508	0.108	0.146
Negative	41	0.519	0.111	0.146
				
*PR status*				
Positive	56	0.503	0.089	0.143
Negative	90	0.525	0.125	0.154

* Denotes a *P*-value of <0.05.
